# The effect of intermittent feeding and cold water on performance and carcass traits of broilers reared under daily heat stress

**DOI:** 10.5713/ajas.19.0980

**Published:** 2020-03-12

**Authors:** Kadir Erensoy, Moise Noubandiguim, Musa Sarıca, Resul Aslan

**Affiliations:** 1 Department of Animal Science, Agricultural Faculty, Ondokuz Mayis University, 55139 Samsun, Turkey; 2 Department of Biology, Faculty of Art and Sciences, Ondokuz Mayis University, 55139 Samsun, Turkey; 3 National High Institute of Sciences and Techniques, Institut National Supérieur des Sciences et Techniques d’Abéché (INSTA), 40823 Abeche, Tchad

**Keywords:** Broiler, Intermittent Feeding, Cold Water, Feed Conversion, Water Intake

## Abstract

**Objective:**

This study aimed to determine the effect of intermittent feeding and cold water on performance and carcass traits in broiler chickens between 4 to 6 wk of age exposed to daily high temperature.

**Methods:**

Broilers were assigned to four treatment groups according to a 2×2 factorial design between 22 to 42 d of age (80 broilers per treatment, 4 replications). Broilers were divided into two main groups as feeding type (*ad-libitum* [AL] and intermittent [IF] for 6 h daily) and sub-groups as water temperature (normal [NW], 24.9°C and cold [CW], 16.4°C). Heat treatment was applied between 11.00 to 17.00 h daily between 22 to 42 d of age.

**Results:**

Live weight at 6th wk was not affected by feeding type and water temperature, but the live weight was significantly higher in IF chickens at the 5th wk (p<0.05). Average weekly gain of IF broiler chickens were higher compared to AL group at 4, 5, and 6 wk of age (p< 0.05). Although feeding type did not affect feed intake in 4 and 5th wk, feed intake was higher in IF chickens at 6th wk (p<0.01). In addition, feeding type and water temperature did not affect feed conversion ratio and interactions were not significant (p>0.05). Water temperature had no significant effect on heart, liver, gizzard, and abdominal fat percentages (p>0.05).

**Conclusion:**

It is concluded that IF increased the average weekly gain in chickens reared under daily heat stress for 6 h between 22 to 42 d of age. IF in hot environmental conditions slightly increased performance without adversely affecting health, welfare, and physiological traits, whereas CW implementation had no significant effect on performance. It can also be said that IF suppresses a sudden increase in body temperature depending on age and live weight.

## INTRODUCTION

High environmental temperatures can cause significant problems in broiler production, especially during summer months. The optimum ambient temperature for poultry is also called thermal neutral zone (18°C to 24°C) and the values above are considered as temperature stress [1]. As the ambient temperature increases, the difference between body temperature decreases and sensible heat loss becomes more difficult [2]. While poultry struggle with high temperature, they also try to keep body temperature within physiological limits by increasing body surface area and respiration rate as well as reducing feed intake and activity [3]. Heat stress causes various harmful effects on physiological, immune, welfare, health, performance and meat quality traits and results in serious economic losses by making internal heat dissipation difficult in poultry [4]. Numerous studies have been carried out to maintain feed efficiency and product quality by reducing body heat production and providing better internal heat dissipation. Genetically developing broiler chickens with featherless neck gene [5], changing feed content [6], limited feeding and lighting programs [7], intermittent feeding (IF) implementation in hot hours [8] and cold water (CW) applications [9] have been reported to reduce metabolic heat production. It has been stated that a daily fasting period of 6 to 8 hours for broiler chickens subjected to heat stress can reduce the fat reserves and provide a profitable production without affecting the mortality rate [10,11].

Water consumption increases at high ambient temperatures [12], but the rise in water temperature also adversely affects performance [13]. It has been reported that as the difference between the temperature of the consumed water and body temperature increases, heat dissipation becomes easier and CW intake improves performance under heat stress conditions [14]. Sudden temperature changes may occur in summer months, especially in non-environmentally controlled houses. During these periods, also depending on the relative humidity, it is not possible to cool by using air velocity, and high mortality rates may occur and decrease the overall performance [15]. The adverse effects of temperature increase on broiler chickens are more pronounced in the second half of the production period (4 to 6 wk), when growth rate and live weight gain are high. This study aimed to determine the effect of IF and CW on performance and carcass traits in broilers between 22 to 42 d of age subjected to daily high temperature.

## MATERIALS AND METHODS

### Chicken and housing material

This study was conducted at Ondokuz Mayis University, Agricultural Faculty experimental farm in June–July 2019. All procedures were approved by Ondokuz Mayis University Ethical Committee for Experimental Animals (30.06.2017; 2017/31).

All eggs were collected and transferred to the hatchery on the same day. After 21 d incubation period, 320 day-old chicks of “*Anadolu-T*” were randomly selected and used for the experiment. The chicks were reared on 8 cm-thick wood shavings litter to by the end of 3th wk with feed and water continuously available during the experimental period (22 to 42 d). Birds were housed in windowed, artificially lighted and ventilated house containing heaters producing hot air automatically. Unit sizes were 1.00×1.50 m, containing one 15 kg capacity tube feeder and 2 nipple drinkers per pen. Economical white bulbs were used for lighting. In order to avoid any effect of intermittent lighting, continuous 24 h lighting was applied during experiment. All chicks were fed *ad-libitum* until the end of the third wk. Water was also given *ad-libitum* during the whole experiment period. The feeds were obtained from a commercial firm and nutritional content of feeds are given in Table 1.

### Experimental design

A total of 320 broiler chickens at the age of 21 days was randomly divided into 4 experimental groups in a 2×2 factorial design that included 2 feeding systems (AL, *ad libitum*; IF, intermittent feeding) and 2 systems of water temperature (NW, normal water; and CW). Feed was provided continuously to AL group for 24 h. The IF group was fasted for 6 h between 11.00 and 17.00 until end of the study. Between these hours, the feeders were taken to a height of about 1 m that chickens could not reach. At the end of this period, the feeders were lowered to the broiler level until the next day fasting period.

In the water temperature treatment, tap water was used for the normal temperature group for 24 h and average of water temperature was 24.9°C during the study. In CW application, the water temperature was kept between 14°C to 18°C with the help of cool-packs for 24 h and the average temperature of water provided was 16.4°C.

The light intensity was 20 to 25 lux in the all chicken pens. At least 33°C to 34°C temperature was provided in the house for the first 3 d and it was gradually decreased by 2°C to 2.5°C per week until 21 d of age. Heat treatment was applied between 11.00 and 17.00 h daily from the beginning of the 4th week to the slaughter age. Actual environmental conditions are given in Table 2.

### Data collection

At the end of the 3th wk, all chickens were tagged with a numbered wing band, individual live weights were taken and 10 males and 10 females were randomly distributed in each pen. The live weight, feed intake and water intake were measured every week at the same time, and the average daily weight gain between each pair of consecutive live weight measurements was calculated for each chick in every weekly interval. A scale with 1 g precision (Jadever, JWQ-6 Digital Precision Scale, Northspring BizHub Industrial Building, Singapore) was used in determination of live weight and feed intake. Water intake was determined by reading the scale on the bucket nipple drinkers located in each pen separately. A 10-liter bucket with 2 nipple drinkers was used as a drinker system per a pen. The amount of water remaining in the drinkers was determined by the scale on the bucket at 9 a.m. every morning and net water intake per chicken was calculated weekly. Mortality was determined daily and weighed in order to calculate the adjusted feed conversion ratio (FCR, kg of feed consumed/kg of body weight). The water temperature was measured 4 times a day (at 9.00, 13.00, 17.00, and 22.00) with a 0.1°C precision liquid thermometer.

The rectal temperature was measured with a manual electronic thermometer (±0.1°C) by inserting into the rectum 2 to 3 cm in depth for each bird. It was carried out twice a week at 14.00 in order to determine the relationship between heat stress and feeding type and water temperature. Each measurement was taken from 8 males and 8 females from each group (64 chickens in total). During the measurement period (30 s) the chicken was gently handled. In the evaluation of the data, the average of the values taken from male and female was used. Dataloggers (Testo 174H, West Chester, PA, USA) were used for monitoring and determination of the house temperature and humidity values every 15 minutes.

Two males and two females close to the mean body weight were selected randomly from each pen and slaughtered after an 8 h fasting period at 42 d of age. Birds were individually weighed and slaughtered by cervical dislocation. Semi-automated equipment was used for scalding (1 min at 56°C), plucking, chilling (in CW, 5 min at 1°C to 5°C), vent-opening, evisceration, and air-chilling (12 h at 4°C). Following air-chilling, abdominal fat was evaluated and recorded as the ratio of fat surrounding abdominal muscles, cloaca and inner organs to live weight. Carcasses were cut into parts according to standard methods, and thigh, breast, neck, wings, back, edible inner organs (heart, liver, and gizzard) and abdominal fat weights were determined. Carcass, edible inner organ and abdominal fat percentages recorded as percentages of cold-carcass weights, and also abdominal fat ratio was calculated by the slaughter weight [16].

### Statistical analysis

The study was designed with a completely randomized design and a 2×2 factorial arrangement (feeding type×water temperature) with 4 replicates. The data were normally distributed and were subjected to statistical analysis using the general linear model of the SPSS (Version 25.0) program. Statistical analyses were used to determine the effects of feeding type, water temperature and the interactions of these factors on broiler chickens performance and carcass traits. Since the interactions are not statistically significant, they are not shown in the tables. The percentages of the studied traits were transformed to arcsine values and then re-transformed to the original values after analysis. Mortality rate was analyzed by chi-square test. Results are given as means and pooled standard error of means. Differences between means were tested with Duncan’s multiple range test at the level of α = 0.05 [17].

## RESULTS

### Growth traits

Live weight at 6th wk was not affected by feeding type and water temperature, but the live weight was significantly higher in IF chickens at the 5th wk (p<0.05). Water temperature did not affect the average weekly gain. IF broiler chickens were higher compared to AL group in terms of average weekly gain at 4, 5, and 6 wk of age (Table 3).

Although feeding type did not affect feed intake in 4th and 5th wk, and it was higher in IF chickens at 6th wk (p<0.01). Although feed intake in chickens that consumed CW was high during the study, but this was not found significant. In addition, feeding type and CW implementation did not affect FCR and interactions also were not significant (Table 4).

Intermittent fed chickens consumed an average of 15.2 mL more water per day between 4 to 6 wk than AL chickens, however this was found to be insignificant (p>0.05). During the study, the water temperature did not affect water intake. The daily water intake of chickens consuming NW and CW was 377.0 and 377.2 mL, respectively. Water intake/feed intake ratio did not change by the treatments, and similar intake occurred in all groups. During the study, water intake/feed intake ratio was found in the range of 2.30 to 2.31 mL for 1 g feed consumption in all groups (Table 5).

### Carcass traits

Although the highest carcass weight was observed in IF chickens (2,026.4 g), the highest carcass yield was 77.2% in chickens that consumed CW; however these differences were insignificant. Water temperature had no significant effect on heart, liver, gizzard, and abdominal fat weights and percentages (p> 0.05). There was no significant effect of water temperature on carcass part weights and ratios (p>0.05). Thigh, breast, wing, back, and neck weights in IF chickens were higher than AL, whereas all carcass part percentages were lower in chickens consuming CW, when compared to NW. However, no significant differences were found in terms of any traits (Tables 6, 7).

### Rectal temperature and mortality

While feeding type and water temperature did not affect the rectal temperature at 4th and 6th wk, significant differences were determined at 5th wk. IF increased rectal temperature at 5 wk of age compared to AL (41.1°C vs 40.9°C; p<0.01). Rectal temperatures of chickens consuming CW and NW were different at the 5 wk of age, and it was determined that CW decreased the body temperature (Table 8).

Higher mortality rate was observed in broiler chickens that consumed AL feed compared to IF (1.7% vs 0.0%). *Ad-libitum* fed broiler chickens were followed by chickens that consumed NW (0.8%). However, none of these differences were significant (p>0.05). There were no deaths in the other groups during the study (Table 8).

## DISCUSSION

High ambient temperatures in broiler production may cause various harmful effects on physiological, immune, welfare, health, performance, and meat quality and serious economic losses may occur [4]. IF chickens use their gizzard and crop to store their feed and maintain their efficiency and performance for extended periods [18]. In the present study, although the live weight of chickens fed intermittent was higher than the AL group at 5 wk of age, this difference was found insignificant at the end of 6th wk. This result was contrary to [19]. Intermittent feeding did not significantly affect live weight gain [18–20]; however in our study, IF chickens gained more live weight compared to AL chickens at 4, 5, and 6 wk of age.

Water temperature may affect the feed, water intake and performance of broiler chickens, especially under hot environmental conditions. When the temperature of consumed water is lower than body temperature, it helps to keep the metabolic temperature lower and increases appetite for water and feed [14]. The CW intake was found to increase the slaughter weight and average weekly gain [12–21]. However, in our study, no significant difference was found between slaughtering weights of broilers consuming NW or CW.

IF and AL implementations did not significantly affect the average daily feed intake of broilers during 4 and 5 wk. However, feed intake was higher in the chickens fed intermittently in the 6th wk [22], and our findings are consistent with this result. Also, broilers fasted for a certain time had a greater tendency to consume feed [22]. Another important point was that this increase in feed intake occurred in the last week and also LW increased. This shows that the amount of feed that cannot be consumed during hot hours is compensated by consuming excessively during cool hours.

The FCR is improved in broiler chickens fed intermittently compared with *ad-libitum* feeding [23,24], however it was determined that there was no significant effect in our study. These results were in line with [25] and who reported that IF does not affect feed conversion ratio. This may be explained by the low tendency of the feed intake in AL groups due to high temperature between 11.00 to 17.00 h. It is seen that there is no significant difference in feed intake between AL and IF chickens between 4 to 6 wk. Cold water intake did not affect feed intake of broilers [24], and similar results were found in our study. However, IF and CW improved FCR during hot hours [24], but these results inconsistent with the our findings.

Although water intake tended to be higher in IF chickens compared to AL, this difference was found insignificant. The water intake/feed intake ratio is 2.02 under normal environmental conditions [26]. However, since the ambient temperatures reached 30°C, this rate increased to 2.30 to 2.31 in our study. Due to the increased ambient temperature, there was an increase in water intake/feed intake ratio, and this increase was not the same as in all treatments. In accordance with our findings, water temperature did not affect amount of water intake [12]. However, there are also studies reporting that the amount of water intake increases as the water temperature increases [27].

The effect of feeding type and water temperature on carcass traits, edible inner organ and abdominal fat levels were insignificant and results were similar to [24]. However unlike our findings, the best carcass yield was determined in chickens fed intermittently for 4 hours [28]. IF increased carcass yield and internal organ ratios compared to AL chickens [29] and these findings were found to be inconsistent with our results. Besides, there are conflicting results between IF and abdominal fat levels. Abdominal fat levels increased as a result of IF [11], while it has been reported that it decreased [29]. However in our findings, the rate of abdominal fat did not change significantly among treatments. Also, it is considered that the similarity of the slaughtering weight did not affect carcass traits significantly. In parallel with this, there was no difference in carcass yield, edible inner organ, and abdominal fat levels.

The amount of breast and thigh meat are composed of 60% to 65% of the carcass. The proportion of carcass parts are mainly dependent on live weight. Some implementations such as IF reduced the amount of breast meat compared to chickens fed *ad-libitum* [30], but the similarity in live weight values in our study did not cause differences breast and thigh percentages among treatments. Consistent with our results, IF treatment in broiler chickens does not affect the percentage of breast, leg and wing [10]. Water temperature had no effect on percentage of carcass components except for the amount of wings, but that the amount of wings of the chickens that consumed CW was lower than drinking NW [12]. In our study, all other carcass parts including the amount of wings did not differ according to water temperature.

IF in broiler chickens helps to maintain performance by preventing excessive body temperature under heat stress conditions [24]. In our study, changes in the performance of IF chickens did not occur at the expected level compared with AL chickens. In terms of rectal temperature, the IF chickens were higher than AL chickens at 5 wk of age. While IF chickens are expected to have a lower body temperature [20–24], the temperature was significantly increased in our study. Rectal temperature and body weight at the 5th wk was also considerably higher in IF chickens. This can be explained by the increase in metabolic heat production with a significant increase in live weight in IF chickens, but the increase in body temperature as a result of poor heat dissipation due to hot ambient conditions. In the 6th wk, there was no difference between IF and AL chickens in terms of rectal temperatures, however some research results show that IF chickens had lower body temperature compared to AL [31,32]. The similarity of the rectal temperatures of IF and AL chickens in the 6th wk shows that these chickens can cope with heat stress and this does not make any difference in their performance. It is thought that the consumption of CW in the 5th week may have prevented the increase in body temperature and this result was in line with [14]. Although CW consumption decreased to rectal temperature slightly (0.2°C) compared with NW, but performance is not affected.. However, IF increased the body temperature by 0.2°C, while AL increased by 0.5°C in chickens during the study. It may be said that IF implementation suppresses the sudden increase in body temperature under hot ambient temperatures.

Actual deaths were within acceptable limits (between 0% to 1.7%) and evaluated as normal. It was found that intermittent feed and CW applications did not affect the mortality rate [12–24].

As a result of the study, it was found that IF increased the average weekly weight gain in broiler chickens reared under daily heat stress for 6 h between 22 to 42 d of age. IF in hot environmental conditions slightly increased performance without adversely affecting health, welfare, and physiological traits, whereas CW implementation had no significant effect on performance. It may also be said that IF suppresses a sudden increase in body temperature depending on age and live weight.

## Figures and Tables

**Table 1:** Nutritional content of feeds used in the study

Nutrients	Starter (1 to 7 d)	Grower 1 (8 to 28 d)	Grower 2 (29 to 35 d)	Finisher (36 to 42 d)
Crude protein (%)	23.00	22.00	21.00	18.00
Metabolizable energy (kcal/kg)	3,000	3,100	3,100	3,100
Crude cellulose (%)	4.00	4.00	4.00	6.00
Crude ash (%)	5.00	5.00	5.00	5.00
Ca (%)	1.00	0.95	0.80	0.80
Phosphorus (%)	0.50	0.50	0.45	0.60
Methionine (%)	1.00	0.45	0.40	0.40
Lysine (%)	1.35	1.20	1.10	1.00

**Table 2:** Temperature and relative humidity levels of the poultry house during 4 to 6 weeks

Weeks	Temperature (°C)		Relative humidity (%)	
	
	Treatment (11.00–17.00 h)	Normal (18.00–10.00 h)	Treatment (11.00–17.00 h)	Normal (18.00–10.00 h)
4th	29.4	24.5	62.8	71.8
5th	29.8	25.2	61.8	70.8
6th	30.8	26.1	59.6	68.1
Mean	30.0	25.3	61.4	70.2

**Table 3:** Effect of feeding type and water temperature on weekly live weight

Treatments	LW (g, week)				AWG (g, week)		
	
	3	4	5	6	4	5	6
Feeding type							
*Ad-libitum*	879.1	1,508.9	2,140.9	2,672.0	629.8	1,264.7	1,790.1
Intermittent	887.5	1,559.5	2,228.3	2,772.7	672.0	1,340.7	1,883.4
Water temp.							
Normal	893.0	1,544.5	2,181.6	2,724.4	651.5	1,288.5	1,831.3
Cold	875.3	1,528.3	2,192.7	2,726.3	653.0	1,319.9	1,846.9
SEM	7.21	14.81	21.74	27.13	8.59	15.51	22.01
p-values							
FT	0.527	0.079	0.040	0.070	0.012	0.016	0.034
WT	0.292	0.741	0.658	0.886	0.751	0.257	0.636
FT×WT	0.085	0.212	0.570	0.617	0.479	0.988	0.244

LW, live weight; AWG, average weekly gain; SEM, standard error of means; FT, feeding type; WT, water temperature.

**Table 4:** Effect of feeding type and water temperature on feed intake and feed conversion ratio

Treatments	FI (g/d, wk)				FCR (g feed/g gain, wk)			
	
	4	5	6	4–6	4	5	6	4–6
Feeding type								
*Ad-libitum*	128.3	167.5	183.6	159.8	1.43	1.85	2.49	1.88
Intermittent	131.9	171.7	193.3	165.6	1.38	1.87	2.52	1.86
Water temp.								
Normal	130.1	168.6	188.3	162.3	1.41	1.85	2.44	1.86
Cold	130.4	170.8	189.2	163.5	1.40	1.80	2.56	1.88
SEM	1.49	2.08	1.88	1.59	0.015	0.028	0.070	0.015
p-values								
FT	0.249	0.288	0.008	0.075	0.089	0.501	0.458	0.707
WT	0.828	0.522	0.629	0.593	0.672	0.370	0.421	0.646
FT×WT	0.594	0.297	0.527	0.648	0.732	0.136	0.130	0.092

FI, feed intake (g/d, wk); FCR, feed conversion ratio (g feed/g gain, wk); SEM, standard error of means; FT, feeding type; WT, water temperature.

**Table 5:** Effect of feeding type and water temperature on water intake

Treatments	WI (mL/d, wk)				WI/FI (mL water/g feed, wk)			
	
	4	5	6	4–6	4	5	6	4–6
Feeding type								
*Ad-libitum*	280.2	379.9	450.8	370.3	2.18	2.27	2.46	2.30
Intermittent	293.0	390.3	470.9	384.7	2.22	2.27	2.44	2.31
Water temp.								
Normal	286.7	386.6	463.4	378.9	2.20	2.29	2.46	2.32
Cold	287.3	384.4	459.9	377.2	2.21	2.25	2.43	2.30
SEM	4.77	6.68	7.00	5.50	0.02	0.02	0.03	0.02
p-values								
FT	0.193	0.439	0.171	0.199	0.405	0.938	0.830	0.821
WT	0.828	0.976	0.939	0.986	0.811	0.479	0.711	0.728
FT×WT	0.370	0.350	0.421	0.320	0.348	0.687	0.195	0.324

WI, water intake (mL/d, wk); WI/FI, water intake/feed intake (mL water/g feed, week); SEM, standard error of means; FT, feeding type; WT, water temperature.

**Table 6:** Effect of feeding type and water temperature on edible inner organs and abdominal fat

Treatments	SW (g)	CW (g)	CDP (%)	Heart (%)	Liver (%)	Gizzard (%)	Abd. fat	

							%1)	%2)
Feeding type								
*Ad-libitum*	2,528.8	1,952.7	77.0	0.64	2.74	1.21	2.60	2.09
Intermittent	2,643.1	2,026.4	76.8	0.64	2.89	1.06	2.87	2.17
Water temp.								
Normal	2,548.3	1,955.4	76.6	0.63	2.90	1.16	2.80	2.13
Cold	2,623.6	2,023.8	77.2	0.64	2.74	1.10	2.66	2.12
SEM	56.87	45.58	0.28	0.02	0.09	0.05	0.19	0.14
p-values								
FT	0.336	0.431	0.545	0.619	0.234	0.149	0.780	0.921
WT	0.524	0.455	0.363	0.594	0.106	0.058	0.973	0.977
FT×WT	0.854	0.699	0.188	0.083	0.228	0.129	0.995	0.854

SW, slaughter weight; CW, cold carcass weight; CDP, cold carcass dressing percentage; SEM, standard error of means; FT, feeding type; WT, water temperature.

1) Percentage of abdominal fat to cold carcass weight.

2) Percentage of abdominal fat to slaughter weight.

**Table 7:** Effect of feeding type and water temperature on carcass parts

Treatments	Percentages (%)				

	Thigh	Breast	Wing	Back	Neck
Feeding type					
*Ad-libitum*	29.4	36.3	10.6	18.6	6.0
Intermittent	28.7	36.5	10.4	18.3	6.1
Water temp.					
Normal	29.3	36.1	10.6	18.2	6.1
Cold	28.2	36.7	10.4	18.7	6.0
SEM	0.31	0.33	0.11	0.32	0.10
p-values					
FT	0.726	0.452	0.189	0.312	0.410
WT	0.726	0.528	0.229	0.804	0.766
FT×WT	0.227	0.592	0.563	0.312	0.099

SEM, standard error of means; FT, feeding type; WT, water temperature.

**Table 8:** Effect of feeding type and water temperature on rectal temperature and mortality

Treatments	Rectal temperature (°C, wk)			Mortality (%)
	
	4	5	6	4 to 6 wk
Feeding type				
*Ad-libitum*	40.9	40.9	41.4	1.7
Intermittent	40.9	41.1	41.1	0.0
Water temp.				
Normal	40.9	41.1	41.2	0.8
Cold	40.9	40.9	41.2	0.4
SEM	0.06	0.05	0.07	0.10
p-values				
FT	0.843	0.004	0.118	0.662
WT	0.560	0.051	0.916	0.662
FT×WT	0.079	0.375	0.787	0.552

SEM, standard error of means; FT, feeding type; WT, water temperature.
